# Prenatal, Perinatal and Postnatal Exposures and Clinical Factors of Food Allergy Prevention: A Review for the First 1000 Days of Life from the SIAIP Primary and Secondary Prevention of Allergic Diseases Commission

**DOI:** 10.3390/nu18172749

**Published:** 2026-08-22

**Authors:** Angela Klain, Salvatore Cascone, Carolina Grella, Valentina Cattivera, Cecilia Fabiano, Francesca Galletta, Sara Manti, Antonio Andrea Senatore, Amelia Licari, Michele Miraglia del Giudice, Gian Luigi Marseglia, Cristiana Indolfi

**Affiliations:** 1 Department of Woman, Child and General and Specialized Surgery, University of Campania ‘Luigi Vanvitelli’, 80138 Naples, Italy; klainangela95@gmail.com (A.K.); salvatore.cascone2@gmail.com (S.C.); michele.miragliadelgiudice@unicampania.it (M.M.d.G.); cristianaind@hotmail.com (C.I.); 2 Department of Pediatrics, University of L’Aquila, 67100 L’Aquila, Italy; valecatt.96@gmail.com (V.C.); fabiano.cecilia@yahoo.it (C.F.); 3 Pediatric Unit, Department of Human Pathology in Adult and Developmental Age ‘Gaetano Barresi’, University of Messina, 98124 Messina, Italy; francygall.92@gmail.com (F.G.); sara.manti@unime.it (S.M.); 4 Pediatric Clinic, Fondazione IRCCS Policlinico, 27100 Pavia, Italy; antonioandrea.senatore01@universitadipavia.it (A.A.S.); amelia.licari@unipv.it (A.L.); 5 Pediatric Unit, Department of Clinical, Surgical, Diagnostic and Pediatric Sciences, University of Pavia, 27100 Pavia, Italy; gianluigi.marseglia@unipv.it

**Keywords:** food allergy, pregnancy, perinatal factors, prevention, children, breastfeeding, siblings, vaccination, environment, microbiome, oral tolerance, eczema

## Abstract

Background/Objectives: Food allergy prevention is no longer understood as the simple avoidance of allergenic foods. The first 1000 days (from conception to, approximately, 24 months of age) include prenatal, perinatal and postnatal windows in which diet, epithelial barrier integrity, microbial ecology, immune maturation and family-level implementation interact. This narrative review was developed as a Commission-proposed SIAIP-oriented clinical framework, rather than a formal guideline or consensus statement. Evidence from guidelines, systematic reviews, randomised trials, birth cohorts and mechanistic studies was organised by developmental window and interpreted according to endpoint validity, confounding, feasibility and clinical readiness. Prenatal factors include maternal allergen non-avoidance, whole-diet quality, micronutrient and fatty acid status, antibiotic exposure, environmental context and maternal microbiome. Perinatal factors include mode of delivery, intrapartum and neonatal antibiotics, gestational maturity, early colonisation, breastfeeding initiation and routine vaccination. Postnatal factors include eczema, skin barrier inflammation, the timing and regularity of allergen introduction, breastfeeding management, infant diet diversity, siblings, daycare, pets, smoke and pollution, physical activity and equitable implementation. The strongest actionable evidence remains eczema-linked risk recognition and timely, sustained oral exposure to peanut and well-cooked egg. Maternal and environmental factors are clinically important, but many are contextual rather than prescriptive; they should guide non-restrictive, non-blaming counselling rather than deterministic risk labelling.

## 1. Introduction

Food allergy (FA) is a heterogeneous immune-mediated condition whose clinical relevance exceeds the acute risk of reactions. It alters nutrition, family behaviour, social participation, quality of life, school routines and healthcare use [1,2,3]. Prevalence estimates vary substantially according to whether an allergy is self-reported, physician-diagnosed, IgE-sensitisation-based or confirmed by oral food challenge. In an updated European meta-analysis, self-reported allergy was several-fold more frequent than challenge-confirmed disease, with differences ranging from approximately 3-fold for egg to 15- to 19-fold for peanut and cow’s milk, a distinction that is central to prevention because both overdiagnosis and delayed recognition can harm families [4].

The decisive shift in prevention came from trials showing that avoidance is not biologically neutral. In the Learning Early About Peanut Allergy (LEAP) trial (*n* = 640), high-risk infants aged 4 to 11 months who consumed peanut regularly had markedly less peanut allergy at 60 months than those assigned to avoidance; the effect persisted after a year of avoidance and into adolescence [5,6,7]. In the Enquiring About Tolerance (EAT) study (*n* = 1303), the early introduction of six allergenic foods in breastfed general population infants was not significant in the intention to treat analysis, but adherence was associated with a lower prevalence of FA (2.4% vs. 7.3%; RR 0.33, 95% CI 0.13–0.83), particularly peanut and egg allergy [8]. These trials establish that timing, dose, route, regularity and feasibility matter in FA prevention.

Pregnancy and birth precede these trials’ intervention windows. Maternal diet, placental transfer of IgG, metabolic and inflammatory state, antibiotics, microbial metabolites, birth mode, human milk and early environmental exposures may shape the context in which food antigens are later encountered [9,10,11,12]. The challenge is to distinguish biologically plausible pathways from clinically proven preventive interventions. For example, maternal IgG immune complexes, short-chain fatty acids and human milk oligosaccharides are compelling mechanisms, but none currently define a validated antenatal prescription or biomarker-guided clinical pathway.

This review therefore uses a temporal structure: prenatal, perinatal and postnatal factors. The first 1000 days are retained as the conventional period from conception to approximately 24 months of age; this manuscript considers the principal evidence relevant to this period, including later follow-up data only when they clarify interventions or exposures originating within this window. Existing prevention guidance provides important recommendations on maternal non-avoidance, breastfeeding support and infant allergen introduction, particularly peanut and egg [13,14,15]. However, these documents are primarily guideline or consensus statements and do not fully address how prenatal, perinatal and postnatal exposures should be integrated into routine counselling across obstetric visits, birth planning, hospital discharge, breastfeeding support, eczema care and complementary feeding. Our contribution is to integrate recent international guidance with an SIAIP-oriented, evidence-weighted counselling framework that distinguishes actionable preventive interventions from contextual risk markers and mechanistic or observational associations. This structure is clinically useful because counselling occurs at different contacts: obstetric visits, birth planning, hospital discharge, breastfeeding support, well-child checks, eczema visits and the start of complementary feeding. It also prevents a common error in prevention writing: mixing maternal diet, birth mode, vaccines, siblings, pets, eczema and early feeding as if all were equally modifiable and equally evidenced. Guidelines support maternal non-avoidance, breastfeeding support and early allergen introduction in appropriate infants, but they do not support maternal blame, vaccine delay, unsafe changes to obstetric care or indiscriminate supplementation [13,14,15,16,17].

## 2. Methods: Literature Search and Evidence Appraisal

A targeted narrative search of PubMed/MEDLINE was conducted and updated through June 2026. The final search date was 30 June 2026. The core search combined terms for FA, prevention and the first 1000 days window: (“food allergy”[Title/Abstract] OR “IgE-mediated food allergy”[Title/Abstract] OR “Food Hypersensitivity”[MeSH Terms]) AND (“prevention”[Title/Abstract] OR “primary prevention”[Title/Abstract] OR “Primary Prevention”[MeSH Terms] OR “oral tolerance”[Title/Abstract]) AND (“pregnancy”[Title/Abstract] OR “Pregnancy”[MeSH Terms] OR lactation[Title/Abstract] OR breastfeeding[Title/Abstract] OR “Breast Feeding”[MeSH Terms] OR infant[Title/Abstract] OR “Infant”[MeSH Terms] OR perinatal[Title/Abstract] OR microbiome[Title/Abstract] OR eczema[Title/Abstract] OR “early allergen introduction”[Title/Abstract]). Because the review addressed multiple prenatal, perinatal and postnatal domains, additional targeted searches were performed for specific exposures, including maternal diet, dietary patterns, vitamin D, omega-3 fatty acids, probiotics, prebiotics, microbiome, short-chain fatty acids, caesarean section, mode of delivery, antibiotics, vaccination, siblings, daycare, pets, tobacco smoke, pollution, eczema, skin barrier and early allergen introduction. Eligible sources were English-language guidelines, systematic reviews, meta-analyses, randomised trials and population-based cohorts addressing prenatal, perinatal or infant exposures and FA or related allergic endpoints; animal and mechanistic studies were retained only when they clarified biologically plausible pathways and were labelled as indirect or preclinical evidence.

Because this is a narrative review and Commission-derived, evidence-informed clinical framework, the evidence supporting each factor was appraised narratively according to four predefined domains: endpoint validity/directness, modifiability during the first 1000 days, susceptibility to confounding or reverse causation and practical clinical translation. These domains were not used to generate formal GRADE ratings, but to structure the interpretation of the evidence and the summary presented in Table 1.

Any terminology used in Table 2 to describe evidence certainty or recommendation strength (e.g., “moderate certainty,” “strong against” or “conditional”) represents a narrative or pragmatic classification developed specifically for this review and should not be interpreted as a formal GRADE certainty rating or recommendation-strength category. Sources were not screened independently in duplicate; selection, prioritisation and interpretation were resolved through author discussion. For evidence appraisal, outcomes were classified as direct when they referred to IgE-mediated, physician-diagnosed or oral-food-challenge-confirmed FA. Outcomes such as sensitisation, eczema, wheeze, asthma, non-IgE-mediated symptoms, immune markers or composite allergic outcomes were considered indirect endpoints and were not interpreted as equivalent to clinical FA prevention.

## 3. Results

The results are presented according to developmental windows; however, this framework is intended to facilitate interpretation rather than to suggest that exposures operate exclusively within discrete periods. Several factors, including diet, microbiome-related factors, antibiotic exposure, breastfeeding and the household environment, may extend across multiple developmental stages. The window-based structure is therefore used to clarify when a given factor can be identified in clinical practice, which preventive actions may be appropriate and the strength of the supporting evidence. This review primarily focuses on the prevention of IgE-mediated FA, for which the strongest intervention evidence is available, particularly from studies on peanut and egg introduction. Evidence concerning sensitisation, eczema, wheeze, asthma, non-IgE-mediated symptoms, microbiome-related markers or composite allergic outcomes is discussed only when it provides relevant mechanistic, contextual or risk-stratification information, and is interpreted as indirect or lower-certainty evidence rather than as direct proof of FA prevention.

### 3.1. Prenatal Factors

#### 3.1.1. Maternal Atopy, Fetal Immune Programming and Placental Antibody Transfer

Prenatal risk begins with the immune and epithelial phenotype inherited and shaped before birth. Family history and maternal atopy are clinically relevant because they increase the prior probability of atopic disease, but they are insufficient as stand-alone predictors of FA [12,18]. The fetal immune environment is influenced by maternal cytokine tone, metabolic status, infection, medication exposure and diet-derived metabolites. Neonatal immunity is developmentally specialised rather than simply immature; type 2-biased responses coexist with regulatory cell expansion, evolving barrier function and later microbial education [19].

Placental IgG transfer is one of the most concrete maternal–fetal pathways. IgG is actively transported through FcRn, and efficiency varies with gestational age, IgG subclass, placental integrity and maternal disease [20,21]. In FA prevention, the relevance is not only passive antimicrobial protection, but also the possibility that allergen-specific IgG immune complexes alter antigen presentation and regulatory programming. This pathway provides a biologically coherent reason not to remove tolerated foods from the maternal diet without indication.

The key mechanistic evidence remains experimental. Ohsaki et al. showed in murine models that maternal IgG–allergen immune complexes could protect offspring from food anaphylaxis, allergen-specific IgE and intestinal mast cell expansion, with FcRn-dependent transfer and induction of allergen-specific regulatory T cells [11]. Yamashita et al. similarly linked maternal antigen-specific IgG immune complexes to suppression of offspring IgE responses [22]. These studies are important because they weaken the rationale for avoidance, but they do not establish a human preventive dose of peanut, egg, milk or wheat during pregnancy.

Maternal age should also be treated as a prenatal contextual variable. Advanced maternal age has been framed as a possible marker of biological, obstetric, environmental and socioeconomic pathways rather than as a single causal mechanism [23].

#### 3.1.2. Maternal Diet: Non-Avoidance, Diversity and Mediterranean-Style Quality

Maternal avoidance of allergenic foods was historically proposed to reduce fetal or milk-borne exposure. Contemporary evidence does not support this approach. The European Academy of Allergy and Clinical Immunology (EAACI) guideline and the American Academy of Allergy, Asthma & Immunology (AAAAI), American College of Allergy, Asthma & Immunology (ACAAI), and Canadian Society of Allergy and Clinical Immunology (CSACI) consensus guidance advises against the exclusion of cow’s milk, egg, peanut, wheat, fish or other tolerated foods for primary prevention because the strategy has not reduced IgE-mediated FA and may compromise nutritional adequacy [13,14]. Garcia-Larsen et al. reached the same broad conclusion in a systematic review of diet during pregnancy and infancy: maternal allergen avoidance was not supported as a preventive intervention [24].

The harm of restriction is not theoretical. Removing dairy, egg, fish, nuts or wheat can reduce protein, calcium, iodine, vitamin D, iron, essential fatty acids and total energy intake, especially when several foods are removed without dietetic support. It also reinforces maternal guilt and can lead to premature breastfeeding cessation when crying, reflux, mild eczema or non-specific stool changes being attributed to milk-borne allergens. Elimination remains appropriate when it is used diagnostically or therapeutically for a symptomatic infant, but it should have a defined target food, nutritional replacement, time limit, endpoint and reintroduction plan.

A stronger prenatal message is therefore not to increase allergen consumption indiscriminately, but rather to avoid the unnecessary exclusion of tolerated allergenic foods while maintaining an overall nutritionally adequate and balanced maternal diet. Systematic reviews of maternal dietary patterns suggest that higher diversity and healthier patterns may be associated with lower allergic outcomes, but the FA-specific signal is smaller and less certain than the signal for wheeze or eczema [25,26]. Chatzi et al. reported that high adherence to a Mediterranean diet in pregnancy was associated with lower persistent wheeze, atopic wheeze and atopy in childhood, although FA was not the primary endpoint [27].

More recent cohorts add detail but not definitive causality. In MEDALLION (analytic sub-cohort, *n* = 430), higher adherence to a Mediterranean diet during pregnancy or lactation was associated with lower odds of offspring FA, but the effect size was modest and residual confounding remains plausible [28]. In Healthy Start (full analytic sample, *n* = 1253), the Maternal Diet Index was associated with lower odds of allergic rhinitis, atopic dermatitis, asthma and wheeze, but not significantly with FA; a later analysis linked maternal diet quality and infant diet diversity with a combined allergic disease outcome [29,30]. Thus, a Mediterranean-style dietary pattern may be recommended primarily for overall maternal and child health and nutritional adequacy, rather than as established interventions for the specific prevention of FA.

#### 3.1.3. Ultra-Processed Foods, Sugar, Pollutants and the Prenatal Exposome

Ultra-processed foods (UPFs), excess free sugars and environmental pollutants belong to the prenatal exposome rather than to classic single-nutrient prevention. A meta-analysis of pregnancy cohorts found only a small association between higher maternal free-sugar intake and offspring asthma risk, with limited evidence for other allergic outcomes [31]. The EAACI task-force report on UPFs reviewed paediatric associations with asthma, rhinitis, eczema and FA, and discussed possible mechanisms including barrier dysfunction, dysbiosis, oxidative stress, advanced glycation end-product signalling and type 2 inflammation [32].

Recent evidence on UPF consumption and paediatric respiratory and allergic diseases has provided a biologically plausible framework linking dietary patterns with immune and inflammatory pathways. However, the available studies predominantly address dietary exposure during childhood and broad allergic phenotypes, rather than the effect of maternal dietary interventions on challenge-confirmed food allergy in offspring [33]. The clinical translation should be proportionate: encourage minimally processed foods, fruit, vegetables, legumes, whole grains and unsaturated fats; reduce sugar-sweetened beverages and frequent highly processed snacks; and avoid fear-based counselling.

Environmental exposures are similarly important but difficult to isolate. Peters et al. summarised a wide range of environmental risk factors for FA, including microbial exposures, pets, siblings, tobacco smoke, pollution, vitamin D, diet and geography [34]. These exposures cluster with income, housing, urbanicity, healthcare access and parental behaviour. Therefore, the prenatal exposome should be used to identify modifiable harms and research priorities, not to create deterministic prenatal risk scores.

#### 3.1.4. Vitamin D, Omega-3 Fatty Acids and Other Maternal Supplements

Vitamin D illustrates the difference between biological plausibility and FA prevention. It influences epithelial, innate and adaptive immune responses and may be relevant to respiratory infections and COVID-19 outcomes in children [35]. However, Zeng et al. reviewed antenatal and early-life vitamin D studies, including randomised trials, and did not find a consistent reduction in eczema or FA after pregnancy supplementation [36]. Omega-3 long-chain polyunsaturated fatty acids are equally nuanced. In DOMInO (allergy follow-up, *n* = 706), maternal supplementation with 900 mg/day *n*-3 LCPUFA from 21 weeks’ gestation reduced egg sensitisation but did not significantly reduce IgE-mediated FA [37]. Meta-analyses have suggested possible effects on eczema, sensitisation, wheeze or asthma, but the evidence is heterogeneous and does not establish broad clinical FA prevention [38,39].

The recent SIAIP position paper on PUFAs appropriately shifts the message away from indiscriminate prescribing and toward risk-stratified, clinically supervised supplementation when dietary intake is low or the risk context supports it [40]. Therefore, omega-3 supplementation should not be presented as a general strategy for FA prevention, but rather as a nutritional intervention with a more consistent rationale in relation to respiratory and inflammatory outcomes, while evidence for challenge-confirmed FA remains limited.

Probiotics, prebiotics and synbiotics require product-specific interpretation. The 2025 Cochrane review reported little or no effect of probiotics on FA by two years and very-low-certainty evidence [41]. Earlier prebiotic evidence was uncertain [42], and the maternal galacto-oligosaccharide/fructo-oligosaccharide (GOS/FOS) trial showed that supplementation can modify maternal and infant microbiota and short-chain fatty-acid profiles without testing clinical FA prevention [43]. The older probiotic meta-analysis suggested effects on eczema depending on timing, but not a reliable effect on all allergic conditions [44].

#### 3.1.5. Maternal Microbiome, Antibiotics, Siblings and Household Ecology

The maternal microbiome is the bridge between diet, medication, household ecology and immune development. Microbial metabolites can influence epithelial integrity, mucus production, dendritic cell phenotype, Treg differentiation, haematopoiesis and gene expression [45,46]. The Barwon Infant Study (*n* = 1064 mothers/1074 infants; case-cohort analysis included 61 infants with clinically proven FA) provides one of the most relevant human signals: in a nested case-cohort analysis, maternal *Prevotella copri* carriage during pregnancy predicted the absence of clinically confirmed IgE-mediated FA in offspring; a larger household size predicted maternal *P. copri* carriage [47].

Sibling exposure is scientifically relevant because a larger household size may reflect greater microbial exchange, daycare contact, shared diet, pet exposure, infection patterns and broader household ecological factors. The classic hygiene hypothesis proposed that a larger household size and older siblings may reduce allergic disease by increasing early microbial exposure [48]. In the FA field, however, siblings should not be presented as a direct intervention. They may reflect a cluster of correlated exposures, including microbial diversity, daycare contact, infections, shared diet, pet exposure, parental behaviour or socioeconomic context. The Barwon *Prevotella* signal supports a household microbiome hypothesis, but it does not mean that family size can or should be manipulated for allergy prevention.

Animal and mechanistic studies explain why the hypothesis is plausible but also why translation should be cautious. A high-fibre maternal diet or acetate exposure can influence offspring allergic airway disease through epigenetic regulation of Foxp3 pathways, but the model addressed airway allergy rather than FA [49]. Maternal *Bifidobacterium bifidum* TMC3115 modified offspring microbiota and immune markers without clearly reducing allergen-specific IgE after sensitisation [50]. Preconception antibiotic depletion changed microbial features across generations but did not deterministically alter FA susceptibility [51].

Antibiotics are therefore best framed as a stewardship issue. The large risk factor meta-analysis found timing-dependent associations between antibiotic exposure and FA, with different estimates for pregnancy, the first month and the first year [18]. A systematic review of early-life antibiotics and childhood FA also supports concern but cannot fully eliminate confounding by indication, infection burden and healthcare-seeking behaviour [52].

### 3.2. Perinatal Factors

#### 3.2.1. Mode of Delivery and Early Colonisation

Vaginal delivery exposes the infant to maternal vaginal and intestinal microbes, whereas caesarean delivery changes the initial colonisation pattern and is often accompanied by perioperative antibiotics. The clinical association with FA, however, is modest. The risk factor meta-analysis reported only a small increase, and the caesarean-specific meta-analysis in children aged 0 to 3 years found higher FA prevalence after caesarean birth than after vaginal birth [18,53].

These data should be interpreted carefully. Caesarean delivery is not randomly assigned; it reflects obstetric indication, maternal disease, gestational age, labour exposure, antibiotic use and neonatal care. Therefore, caesarean birth should be documented as a contextual factor but never presented as a preventable cause of FA for an individual child. Allergy prevention must not compromise obstetric safety.

Microbiome studies support biological plausibility. Infant gut microbiota composition in the first year has been associated with food sensitisation, and reduced microbial richness or altered *Enterobacteriaceae/Bacteroidaceae* patterns have been reported in early-life allergy research [54]. These findings do not justify routine microbiome testing, vaginal seeding or commercial microbial replacement for FA prevention. They do justify antimicrobial stewardship, breastfeeding support where possible and longitudinal research that links microbial function to oral food challenge outcomes.

#### 3.2.2. Gestational Maturity, Neonatal Care and Antibiotics

Prematurity, low birth weight and neonatal intensive care are not classic allergy prevention targets, but they can alter feeding trajectories, microbial assembly, antibiotic exposure, skin integrity and family anxiety. A premature infant may require delayed or adapted complementary feeding according to developmental readiness, but prematurity alone should not lead to the prolonged avoidance of allergenic foods once oral feeding is safe [14,15].

Perinatal antibiotics deserve a separate discussion from prenatal antibiotics because the first days of life are a dense period of microbial assembly. The evidence linking antibiotics to FA is associative and confounded, but repeated or unnecessary exposure should still be avoided because stewardship has benefits beyond allergy [18,52].

The perinatal visit is also an opportunity to prevent later implementation failure. Families can be told before discharge that eczema appearing very early should prompt active treatment and, in moderate-to-severe cases, allergy-informed feeding planning. This is more useful than assigning risk based only on birth mode or antibiotic history.

#### 3.2.3. Breastfeeding and Human Milk as a Perinatal–Postnatal Immune Interface

Breastfeeding begins perinatally and continues postnatally, so it is best treated as an interface rather than a single exposure. Human milk contains secretory IgA, IgG, IgM, cytokines, growth factors, leukocytes, extracellular vesicles, human milk oligosaccharides (HMOs), microbes, microbial products, metabolites and small quantities of maternal dietary antigens [55,56]. Among these components, HMOs are structurally diverse glycans that may influence mucosal immune development by shaping the infant gut microbiota, supporting epithelial barrier maturation, limiting pathogen adhesion through soluble decoy effects and modulating innate as well as adaptive immune signalling. HMO profiles have been associated with cow’s milk allergy risk in small cohorts, but the current evidence does not establish a specific protective HMO pattern or an HMO-based strategy for FA prevention [56,57,58]. Thus, these mechanisms should be interpreted as biologically plausible but not clinically prescriptive.

This composition varies with lactation stage, maternal genetics, secretor status, diet, infection, geography, atopy and collection methods. These biological components provide plausible mechanisms through which human milk may influence immune development, but their individual contribution to FA prevention remains uncertain. Duration and exclusivity alone cannot capture this biology. Clinical outcome evidence therefore remains heterogeneous and should not be reduced to breastfeeding duration or exclusivity alone.

The most clinically useful point is that reflex maternal elimination is usually unjustified. Gamirova et al. reviewed studies measuring cow’s milk, egg, peanut and wheat proteins in human milk and estimated that most concentrations were below eliciting doses expected to trigger immediate IgE-mediated reactions in allergic individuals [59]. This does not exclude rare reactions or non-IgE-mediated disease, but it means that crying, reflux, mild eczema or stool changes in a thriving infant should not automatically lead to multi-food maternal restriction. Maternal peanut intake during lactation provides a further example of the need for caution. In a secondary analysis of the LEAP avoidance arm, moderate maternal peanut consumption while breastfeeding was associated with less peanut sensitisation, but maternal intake was not randomised and the clinical allergy endpoint was not definitively reduced [60]. The finding supports the avoidance of unnecessary maternal dietary restriction, but does not establish that maternal peanut consumption during lactation prevents clinical peanut allergy or defines a preventive dose–response relationship.

Small mechanistic studies provide plausible tolerance pathways. In term neonates born by caesarean section (*n* = 38), exclusive breastfeeding was associated with a higher proportion of circulating regulatory T cells and lower responses to non-inherited maternal antigens [61]. HMO profiles have also been linked to cow’s milk allergy risk in small cohorts, and reviews have highlighted HMOs as candidate immune-modifying components [57,58]. Human milk also contains small quantities of maternal dietary antigens, including cow’s milk, egg, peanut and wheat proteins. However, although these proteins may be biologically relevant to immune development, current evidence does not establish that their presence is generally protective or that there is a preventive dose–response relationship. These observations are important for research, but they are not yet sufficient for HMO-based product prescribing or for recommending specific maternal dietary exposures during lactation as FA prevention strategies [60]. Recent meta-analytic evidence further supports a cautious interpretation of breastfeeding. Ding et al. synthesised cohort studies on breastfeeding, FA and allergic rhinitis and reported heterogeneous associations, reinforcing that breastfeeding should be protected for its broad maternal–infant benefits but should not be overpromised as a stand-alone or complete prevention strategy for IgE-mediated FA [62].

Human milk should be interpreted as a dynamic biological matrix rather than a binary exposure. Secretory IgA, IgG immune complexes, cytokines, extracellular vesicles, HMOs, microbial metabolites and low-dose food antigens may interact with infant epithelial and mucosal immune development. However, their concentration and function vary according to lactation stage, maternal diet, infection, secretor status, geography and sampling methods. This variability may explain why duration-only analyses often produce inconsistent findings regarding allergy outcomes.

For counselling, the message should be protective rather than restrictive. Breastfeeding support and maternal dietary adequacy should be prioritised together. When symptoms are reproducible and temporally related to feeding, maternal elimination can be used as a structured diagnostic trial; when symptoms are non-specific, broad elimination risks nutritional harm and breastfeeding interruption without proving causality.

#### 3.2.4. Vaccination in the Perinatal and Early Infancy Pathway

Vaccination must be discussed because families frequently ask whether vaccines can worsen allergy risk. Current evidence does not support delaying routine vaccination as a strategy for FA prevention. Navaratna et al. conducted a systematic review and meta-analysis of childhood vaccination and allergy and did not provide evidence that vaccine delay is a justified allergy prevention strategy [63].

The clinical framing is essential. Vaccines are not an FA prevention intervention, but neither are they a modifiable exposure to remove for allergy prevention.

### 3.3. Postnatal Factors

#### 3.3.1. Eczema, Skin Barrier Dysfunction and Transcutaneous Sensitisation

Postnatal prevention begins with the skin because eczema is the most reproducible clinical marker of FA risk. In HealthNuts (*n* = 4453 infants in this analysis), challenge-proven peanut, egg or sesame allergy affected one in five infants with eczema compared with one in twenty-five without eczema. Infants with eczema before 3 months requiring prescribed topical corticosteroids had particularly high FA prevalence [64]. The large 2026 risk factor meta-analysis confirmed eczema, transepidermal water loss and filaggrin-related barrier factors as major risk signals [18].

The clinical implication is not merely dermatological. Inflamed skin can permit environmental exposure to food proteins in a pro-sensitising context, whereas gastrointestinal exposure in an appropriate dose and regular pattern can promote tolerance. Therefore, eczema care and feeding advice must be connected. A child with early, persistent or moderate to severe eczema should not simply receive emollient advice; the care pathway should include risk assessment, active anti-inflammatory treatment and timely planning for peanut and cooked egg introduction.

Routine emollient prevention has not solved this problem. PreventADALL tested early food intervention and skin emollients in a factorial design and found that food intervention reduced FA, whereas emollient skin intervention did not [65]. The Cochrane individual participant data meta-analysis of skincare interventions similarly did not support routine emollients as a population-level strategy to prevent eczema or FA [66]. These findings distinguish prophylactic emollient use from the active treatment of established eczema. Once eczema is present, appropriate anti-inflammatory treatment and skin barrier management remain essential and may reduce skin inflammation and transcutaneous sensitization, providing a different clinical rationale from universal emollient prophylaxis. The treatment of established eczema remains essential as standard clinical care, but this should not be interpreted as evidence that active eczema treatment independently prevents FA. The role of eczema in this context should therefore be considered in three distinct ways: eczema is an important marker for identifying children at increased risk of FA; treatment of established eczema is essential clinical care; and skin-directed interventions specifically aimed at preventing FA remain an area of limited evidence. Thus, while appropriate eczema management may reduce skin inflammation and transcutaneous sensitisation and has a strong clinical rationale, it has not been independently proven to prevent FA. Universal emollient prophylaxis is a different question and is not supported as a population-level FA prevention strategy.

Recent paediatric atopic dermatitis literature strengthens this skin barrier framing. Reviews on biomarkers in childhood atopic dermatitis, atopic march and biological treatments emphasise that eczema severity reflects an integrated epithelial, microbial and type 2 inflammatory phenotype rather than a cosmetic skin problem [67,68,69,70].

In children with moderate–severe atopic dermatitis, studies on dupilumab and *Staphylococcus aureus* colonisation further illustrate the interaction between barrier repair, microbial ecology and inflammatory control [68,69,71]. These treatment studies do not prove primary FA prevention and should not be interpreted as evidence that active eczema control independently prevents FA. Rather, they support the clinical principle that early, active eczema management is essential standard care and may form part of a broader allergy prevention framework, while the specific preventive effect on FA remains unproven.

#### 3.3.2. Timing, Dose and Regularity of Oral Allergen Introduction

The strongest preventive evidence is postnatal and oral, but it is not uniform across foods [72]. LEAP demonstrated that sustained peanut consumption in high-risk infants produced large reductions in peanut allergy, and follow-up showed that protection persisted beyond the intervention period and into adolescence [5,6,7]. Its lesson is not simply “early peanut”; it is a structured introduction in a defined risk phenotype, with ongoing ingestion and clear safety pathways [14,73].

EAT extended the question to general population breastfed infants and multiple foods [8]. The trial’s intention to treat result was not significant, whereas the per protocol analysis showed lower food allergy among adherent infants (2.4% vs. 7.3%; RR 0.33, 95% CI 0.13–0.83), particularly peanut and egg allergy [8]. This distinction matters clinically: advice that families cannot implement does not prevent disease. The introduction must be developmentally appropriate, culturally feasible and repeated often enough to maintain exposure [8,14,72].

PreventADALL (*n* = 2397 newborn infants) strengthened the evidence for early food intervention in a general population context, with lower FA after the introduction of peanut, egg, milk and wheat [65]. Scarpone et al. (23 trials; 13,794 participants) then synthesised randomised trials and found that earlier egg introduction reduced egg allergy, earlier peanut introduction reduced peanut allergy and multiple food introduction reduced overall IgE-mediated FA, with the strongest certainty for peanut and egg; however, adherence and withdrawal were important limitations, and the certainty for cow’s milk and wheat was much lower than for peanut and egg [72].

For peanut, current guidance is the most operational. The NIAID Addendum Guidelines recommend considering peanut introduction at 4–6 months in infants with severe eczema and/or egg allergy, after other solid foods have shown developmental readiness and after peanut-specific IgE and/or skin prick testing when clinically indicated [73]. Infants with mild to moderate eczema may introduce peanut-containing foods around 6 months, whereas infants without eczema or food allergy may introduce peanut in an age-appropriate manner according to family and cultural practice [73]. When peanut is introduced, it should be offered in safe, non-choking forms, such as smooth peanut-containing preparations diluted into tolerated foods, and continued regularly [14,73]. The NIAID implementation dose is approximately 6–7 g of peanut protein per week, divided over three or more feedings [73].

For hen’s egg, the evidence and practical guidance are related but not identical to peanut. EAACI suggests introducing well-cooked hen’s egg, but not raw or uncooked pasteurised egg, as part of complementary feeding [13]. The most effective window in the available trials appears to be approximately 4–6 months, once the infant is developmentally ready [13,72]. Practical guidance refers to small amounts of well-cooked egg given repeatedly, for example about half of a well-cooked small egg twice weekly or an equivalent well-baked form [13]. This recommendation should therefore be framed as cooked egg introduction during complementary feeding, not as unrestricted early exposure to raw or lightly cooked egg.

Evidence for cow’s milk, wheat and multiple food introduction is less certain and less operational [72]. Milk, wheat and other tolerated allergens should be introduced as part of a varied complementary diet in safe age-appropriate forms, but they should not be presented with the same certainty, timing precision or dose specification as peanut and well-cooked egg [14,65,72].

Accordingly, in this review “early introduction” means introduction during the complementary feeding window, once developmental readiness is present, with food-specific attention to risk, form, dose, regularity and safety [13,14,15,73]. Infants with previous immediate reactions to a food, known FA or severe uncontrolled eczema should receive individualised allergy assessment before the unsupervised introduction of the suspected food [14,73]. Conversely, indiscriminate allergy panels and the unnecessary avoidance of tolerated foods should be avoided because they may increase dietary restriction without improving prevention [14,73].

Guidelines vary in detail but converge on avoiding delayed introduction and avoiding maternal restriction as a substitute for infant feeding guidance. The AGREE review of infant feeding prevention guidelines showed substantial variation in quality and content [74]. The trial’s intention to treat result was not significant, whereas the per protocol analysis showed lower FA among adherent infants (2.4% vs. 7.3%; RR 0.33, 95% CI 0.13–0.83), particularly peanut and egg allergy [8].

#### 3.3.3. Infant Diet Diversity, Family Diet and Nutritional Continuity

Postnatal diet diversity is the infant counterpart of maternal diet quality. The Venter analyses suggest that maternal diet quality and infant dietary diversity are linked to allergic outcomes, but the effect is not specific enough to prescribe a proprietary score [29,30]. The practical message is continuity: families should move from a varied maternal diet to a varied complementary diet, including common allergens in safe forms and textures.

This matters in Italy because dietary advice must be culturally realistic. Peanut may not be a traditional infant food, egg may be introduced in different culinary forms and families may fear choking or allergic reactions. FA prevention counselling during complementary feeding should therefore specify forms such as smooth peanut-containing preparations and well-cooked egg, avoid whole nuts in infants and emphasise regular ingestion after successful introduction.

Infant diet diversity has a broader evidence base than the maternal index studies alone. Roduit et al. reported that greater food diversity during the first year of life was inversely associated with allergic diseases, while Nwaru et al. found associations between food diversity in infancy and a lower risk of childhood asthma and allergic outcomes [75,76]. This concept is further supported by the EAACI position paper on diet diversity and allergy, by a prospective cohort of 2251 mother–infant pairs showing an inverse association between higher food diversity in the first year and allergic outcomes in the second year of life, and by a recent systematic review in which all high-quality cohort studies assessing food allergy reported protective associations, although definitions and endpoints were heterogeneous [77,78,79]. Overall, these data support infant diet diversity as a developmental feeding principle, but the evidence should be interpreted cautiously because many outcomes were observational, composite or not based on challenge-confirmed IgE-mediated FA.

The practical consequence is that early allergen introduction should not be isolated from the overall quality and diversity of complementary feeding. Families need examples that fit local diets: well-cooked egg incorporated into usual meals, safe peanut-containing preparations where culturally acceptable, legumes, cereals, fruit, vegetables, fish and dairy when tolerated. Repeated ingestion after successful introduction is as important as the first exposure, because tolerance is a maintained biological state rather than a single event.

Family diet is also relevant. Infants do not eat in isolation: parental food habits, fear of reactions, cost, cooking skills, childcare routines and access to dietetic advice determine whether prevention advice becomes regular intake.

#### 3.3.4. Use of Supplements During Infancy

The use of dietary supplements during infancy should also be considered among postnatal factors potentially relevant to food allergy prevention. In the United States, the use of supplements such as vitamin D, dietary fibre and probiotics during infancy is relatively common. However, the evidence regarding their role in preventing FA remains heterogeneous, and current data do not support routine supplementation as a universal strategy for FA prevention [36,41,42,43,44]. Vitamin D supplementation may be recommended according to nutritional status and national guidelines, while the potential preventive effects of probiotics and other supplements require further investigation. Therefore, supplementation should be considered on an individual basis, taking into account the infant’s nutritional needs and clinical context, rather than as a substitute for established preventive strategies such as appropriate complementary feeding and the timely introduction of common allergens.

#### 3.3.5. Siblings, Daycare, Pets, Smoke, Pollution and the Lived Environment

Postnatal environmental exposures should be interpreted within a balanced framework that considers microbial exchange, epithelial barrier injury, immune metabolic context and exposure clustering. Siblings and daycare may increase microbial exchange; pets may alter indoor microbial communities; smoke and air pollution can injure epithelial barriers and promote inflammation; and diet as well as outdoor activity shape immune metabolic context. Peters et al. reviewed these environmental risk factors and emphasised heterogeneity, exposure clustering and the difficulty of causal inference [34].

Pet exposure is a good example. In the Japan Environment and Children’s Study (analytic sample, *n* = 66,215 children), fetal or infancy exposure to household pets was associated with food allergy outcomes, with differences by animal type and food allergen. Dog exposure was associated with a lower risk of egg, milk and nut allergy, whereas cat exposure was associated with a lower risk of egg, wheat and soybean allergy; conversely, fetal hamster exposure was associated with a higher risk of nut allergy [80]. However, the observational nature of the evidence means that pet exposure should not be translated into advice to acquire or remove pets solely to prevent FA. Rather, it should be considered as part of the broader household environment.

Siblings should be handled similarly. Although household size has been linked to allergy risk and maternal microbial profiles associated with lower offspring FA risk [47,48], siblings are not a modifiable preventive intervention.

The summary of the available evidence has been provided in Table 1. Table 1.

**Table 1:** Evidence appraisal of prenatal, perinatal and postnatal factors discussed in relation to IgE-mediated FA prevention and related allergic outcomes during the first 1000 days.

Window	Evidence Source	Population/Design/Comparator	Exposure or Intervention	FA Endpoint/Directness	Principal Quantitative Result	Certainty/Key Limitation/Clinical Translation
General	Spolidoro et al. (2023) [4]	110 European studies; comparator was method of ascertainment.	Epidemiology of eight major food allergies.	Self-reported FA, physician-diagnosed FA, sIgE/SPT sensitisation, clinical history/OFC and challenge-positive FA.	Lifetime self-reported FA 19.9% (95% CI 16.6–23.3); point self-reported FA 13.1% (11.3–14.8); sIgE 16.6% (12.3–20.8); SPT 5.7% (3.9–7.4); challenge-positive FA 0.8% (0.5–0.9).	Moderate risk of bias in most studies; endpoint definition strongly changes prevalence estimates and must be explicit.
Prenatal/infancy	Garcia-Larsen et al. (2018) [24]	260 milk-feeding studies (964,143 participants) and 173 other diet studies (542,672 participants), including 80 trials; comparators varied by diet/supplement exposure.	Maternal and infant diet, avoidance diets and supplements.	FA, sensitisation, eczema, asthma/wheeze and autoimmune outcomes; many endpoints indirect for FA.	Maternal allergen avoidance was not supported. Probiotics in late pregnancy/lactation reduced eczema (RR 0.78, 95% CI 0.68–0.90); fish oil reduced egg sensitisation (RR 0.69, 95% CI 0.53–0.90), not clinical FA.	Moderate for some surrogate outcomes but indirect for FA prevention; heterogeneous interventions and outcome definitions.
Prenatal	Tan et al. (2025) [25]	28 papers reporting 29 dietary pattern, diet index or diversity measures; mainly observational comparisons of higher vs. lower pattern adherence.	Maternal dietary patterns during pregnancy.	Offspring allergy outcomes, including FA, eczema, wheeze/asthma and sensitisation; FA often not challenge-confirmed.	Diet diversity during pregnancy showed a small association with lower FA risk (OR 0.95, 95% CI 0.92–0.99); pro-inflammatory diet was associated with asthma/wheeze in early childhood.	Low certainty for FA; residual confounding and heterogeneous scores mean this supports healthy diet counselling, not proven FA prophylaxis.
Prenatal	Venter et al./EAACI review (2020) [26]	95 papers from 50 studies; 11 RCTs, 30 cohorts and other observational designs; supplement or diet exposure compared with placebo/no exposure or lower exposure.	Dietary factors during pregnancy.	Asthma/wheeze, AD, FA and sensitisation; FA-specific endpoints limited.	Vitamin D supplementation reduced asthma/wheeze (OR 0.72, 95% CI 0.56–0.92), but neither vitamin D nor omega-3 changed AD or FA risk; omega-3 trends for asthma/wheeze and rhinitis were non-significant.	Indirect for FA prevention; supports non-restrictive diet quality and caution with supplementation claims.
Prenatal	Gupta et al. (2022) [31]	5 articles from 4 cohort studies; higher vs. lower maternal free-sugar intake.	Maternal free-sugar intake.	Asthma and other allergic outcomes, including FA in limited analyses; largely parent-reported/observational.	Higher free-sugar intake was associated with offspring asthma (OR 1.07, 95% CI 1.00–1.14; *I*^2^ = 0%). Associations with FA and other allergic outcomes were less robust.	Low certainty and indirect for FA; supports general maternal–child health advice rather than FA-specific prevention.
Prenatal/early life	Zeng et al. (2023) [36]	43 articles including 6 RCTs; vitamin D supplementation or measured vitamin D status compared with placebo/no supplement or lower status.	Antenatal or early-life vitamin D.	Eczema and FA; direct FA evidence limited.	Pregnancy vitamin D supplementation did not reduce eczema (OR 0.85, 95% CI 0.67–1.08; 4 RCTs, *n* = 2074). Three RCTs reported null FA findings. Cord blood vitamin D was associated with lower eczema (OR 0.89 per 10 nmol/L, 95% CI 0.84–0.94).	No consistent FA prevention; observational status data are vulnerable to confounding.
Prenatal	Best et al. (2016) [38]	13 publications from 10 cohort studies and 7 publications from 5 RCTs; higher omega-3 exposure or supplementation vs. lower exposure/control.	Omega-3 LCPUFA intake or supplementation in pregnancy.	IgE-mediated allergic disease and sensitisation; clinical FA evidence limited.	RCT meta-analysis reduced atopic eczema (RR 0.53, 95% CI 0.35–0.81), any positive SPT (RR 0.68, 95% CI 0.52–0.89), egg sensitisation (RR 0.55, 95% CI 0.39–0.76) and any food sensitisation (RR 0.59, 95% CI 0.46–0.76).	Mostly indirect/sensitisation outcomes; does not establish broad prevention of challenge-confirmed FA.
Prenatal/lactation/infancy	Bärebring et al. (2022) [39]	9 RCTs; *n*-3 supplementation in pregnancy, lactation or infancy vs. placebo/control.	Long-chain *n*-3 fatty acids.	Asthma/wheeze, eczema, FA and sensitisation.	Pregnancy supplementation reduced asthma/wheeze (RR 0.62, 95% CI 0.34–0.91; *I*^2^ = 67.4%), but not FA (RR 0.63, 95% CI 0.06–1.20; 4 studies).	FA evidence inconclusive; use for nutritional adequacy/risk-stratified contexts, not universal FA prophylaxis.
Prenatal/infancy	Wang et al./Cochrane (2025) [41]	24 studies; 7077 mother–infant pairs; probiotics/synbiotics vs. placebo/no supplement in the first 6 months.	Infant probiotics or synbiotics.	IgE-mediated FA by 2 years and other allergic outcomes.	Little or no effect on IgE-mediated FA by 2 years (RR 1.12, 95% CI 0.57–2.20; 3 studies, *n* = 857; very-low-certainty evidence).	Very low certainty for FA; routine probiotic use for FA prevention is not supported.
Prenatal/infancy	Cuello-Garcia et al. (2017) [42]	22 RCTs; no studies in pregnant or breastfeeding women; infant prebiotic supplementation vs. placebo/no supplement.	Prebiotics.	Eczema, wheeze/asthma and FA; FA endpoint sparse.	Eczema RR 0.68 (95% CI 0.40–1.15), wheeze/asthma RR 0.37 (0.17–0.80), FA RR 0.28 (0.08–1.00).	Very low certainty due to risk of bias and imprecision; not sufficient for routine FA prevention.
Prenatal/perinatal/infancy	Cuello-Garcia et al. (2015) [44]	29 RCTs; probiotics to pregnant women, breastfeeding mothers and/or infants vs. placebo/no probiotics.	Probiotics for allergy prevention.	Eczema and other allergic conditions; FA-specific protection not shown.	Reduced eczema when used in late pregnancy (RR 0.71, 95% CI 0.60–0.84), during breastfeeding (RR 0.57, 95% CI 0.47–0.69) or in infants (RR 0.80, 95% CI 0.68–0.94).	Low/very low certainty and indirect for FA; no clear prevention of other allergies or FA.
Cross-window risk	Islam et al. (2026) [18]	176 studies assessing 342 risk factors for childhood FA; exposed vs. unexposed children.	Risk factors including eczema, delayed allergen introduction, antibiotics, family history and caesarean delivery.	Food allergy incidence/risk; endpoint definitions varied, with some challenge-confirmed data.	Higher risk with eczema in first year (OR 3.88), increased TEWL (OR 3.36), peanut introduction after 12 months (OR 2.55), antibiotics in first month (OR 4.11), maternal FA history (OR 1.98) and caesarean delivery (OR 1.16).	Useful for risk recognition; many factors are observational risk markers, not preventive targets.
Perinatal/infancy	Netea et al. (2019) [52]	6 studies, 2 prospective and 4 retrospective; antibiotic-exposed vs. unexposed infants.	Early-life antibiotic exposure.	Childhood FA; observational definitions varied.	Four of six studies found significant associations; two suggested a dose–response relationship. No pooled estimate was reported.	Low certainty; confounding by indication, infection burden and healthcare use remains major.
Perinatal	Yang et al. (2023) [53]	9 cohort studies; caesarean-born vs. vaginally born children aged 0–3 years.	Caesarean delivery.	FA prevalence in early childhood; definitions varied.	FA prevalence was 7.8% in caesarean-born vs. 5.9% in vaginally born children (OR 1.45, 95% CI 1.03–2.05), with parental allergy history OR 2.60 (95% CI 1.28–5.27).	Observational and confounded by indication; not a modifiable FA prevention target.
Lactation	Gamirova et al. (2022) [59]	32 studies measuring cow’s milk, egg, peanut and wheat proteins in human milk.	Food proteins in human milk after maternal intake.	Estimated probability of IgE-mediated reactions through breastfeeding, not prevention.	Most concentrations were below ED01; estimated probability of an IgE-mediated reaction through breast milk was ≤1:1000 for cow’s milk, egg, peanut and wheat.	Supports avoiding unnecessary maternal restriction; does not establish a protective dose response.
Postnatal/lactation	Ding et al. (2024) [62]	68 observational studies; 772,142 children; breastfeeding categories compared with shorter/no breastfeeding.	Breastfeeding duration and pattern.	FA and allergic rhinitis; heterogeneous, mostly observational outcome definitions.	Breastfeeding >6 months was associated with lower allergic rhinitis risk (OR 0.88, 95% CI 0.79–0.98), but FA-specific results were inconsistent, including higher FA odds in one analysis (OR 1.69, 95% CI 1.27–2.25).	Substantial confounding/reverse causation; support breastfeeding for broad health benefits, not stand-alone FA prevention.
Postnatal	Navaratna et al. (2021) [63]	35 cohort publications and 7 RCT publications; vaccinated vs. unvaccinated/different timing.	Childhood vaccination.	Allergic outcomes including eczema, asthma, sensitisation and FA.	Early BCG vaccination reduced eczema in RCTs (RR 0.83, 95% CI 0.73–0.93), but not FA or asthma. Measles vaccination in cohorts was associated with lower eczema (RR 0.65) and asthma (RR 0.78).	Indirect for FA; no evidence supports vaccine delay for allergy prevention.
Postnatal	Kelleher et al./Cochrane (2021) [66]	33 identified RCTs; skincare interventions in healthy infants vs. standard care/no intervention; 7 trials (*n* = 3075) for eczema and 1 trial (*n* = 996) for IgE-mediated FA.	Routine emollient or skincare intervention.	Eczema, IgE-mediated FA and food sensitisation.	Skincare did not reduce eczema (RR 1.03, 95% CI 0.81–1.31). It was unclear whether it increased IgE-mediated FA (RR 2.53, 95% CI 0.99–6.47; very low certainty).	Does not support population-level emollient prophylaxis; distinct from treating established eczema.
Postnatal	LEAP trial (2015) [5]	640 high-risk infants aged 4–11 months with severe eczema and/or egg allergy; peanut consumption vs. avoidance.	Sustained peanut consumption.	Challenge-confirmed peanut allergy at 60 months; direct IgE-mediated FA outcome.	In baseline SPT-negative infants, peanut allergy was 13.7% with avoidance vs. 1.9% with consumption; in baseline SPT-positive infants, 35.3% vs. 10.6%.	High certainty for peanut in defined high-risk phenotype; requires safety assessment and sustained ingestion.
Postnatal	EAT trial (2016) [8]	1303 exclusively breastfed general population infants; early introduction of six foods from 3 months vs. standard introduction around 6 months.	Multiple allergenic foods: milk, peanut, cooked egg, sesame, whitefish and wheat.	Food allergy to one or more intervention foods between 1 and 3 years; direct, challenge-based assessment.	Intention to treat difference was not significant (5.6% vs. 7.1%). Per protocol FA was lower with adherence (2.4% vs. 7.3%; RR 0.33, 95% CI 0.13–0.83), especially peanut and egg.	Implementation/adherence is the key limitation; supports feasible, sustained exposure rather than simple advice.
Postnatal	PreventADALL trial (2022) [65]	2397 newborn infants; factorial cluster RCT; food intervention and/or skin emollients vs. no corresponding intervention.	Small repeated tastes of peanut, cow’s milk, wheat and egg from 3 months; skin emollients from 2 weeks.	Allergy to intervention foods at 36 months; direct clinical food allergy endpoint.	Food allergy occurred in 2.3% no intervention, 3.0% skin, 0.9% food and 1.2% combined groups; food intervention vs. no food intervention OR 0.4 (95% CI 0.2–0.8); NNT 63.	General population RCT; low event rate and adherence/practicality considerations.
Postnatal	Scarpone et al. (2023) [72]	23 RCTs; 13,794 participants; earlier vs. later introduction of allergenic foods.	Timing of allergenic food introduction.	IgE-mediated FA at 1–5 years; direct for peanut/egg and multiple food outcomes.	Earlier egg reduced egg allergy (RR 0.60, 95% CI 0.46–0.77; high certainty); earlier peanut reduced peanut allergy (RR 0.31, 95% CI 0.19–0.51; high certainty); multiple food introduction reduced any FA (RR 0.49, 95% CI 0.33–0.74) but increased withdrawal (RR 2.29, 95% CI 1.45–3.63).	Strongest certainty for peanut and egg; adherence and food-specific implementation limit broader extrapolation.
Postnatal implementation	Vale et al. (2021) [74]	1121 records plus reference checking; 28 infant feeding FA prevention guideline/advice documents identified.	Guideline quality and operational detail.	Guideline recommendations rather than clinical FA endpoint.	Eight of ten documents titled ‘guidelines’ met AGREE II quality threshold; 0 of 18 advice documents did. Variation was greatest for breastfeeding and timing of solids.	Supports clearer, operational counselling tools and explicit evidence to statement links.
Postnatal diet	Lv et al. (2024) [79]	15 high-quality publications; mainly cohort studies; higher vs. lower infant diet diversity.	Diet diversity during infancy.	Atopic outcomes including FA, food sensitisation, AD, asthma and rhinitis; FA definitions heterogeneous.	All high-quality cohort studies assessing FA reported protective associations (5/5); asthma 4/6, food sensitisation 3/5 and AD 3/5 showed favourable signals.	Observational/indirect; supports diet diversity as feeding principle, not a precise FA prevention dose.
Postnatal environment	Okabe et al. (2023) [80]	97,413 mother–child pairs in JECS; pet exposure during fetal life/infancy vs. no exposure.	Household pets.	Parent-reported food allergy outcomes by age 3; observational.	Dog or cat exposure was associated with lower incidence of all FA; dog exposure was linked to lower egg, milk and nut allergy, cat exposure to lower egg, wheat and soybean allergy, while hamster exposure was linked to higher nut allergy.	Contextual, confounded and culture-specific; do not prescribe acquiring/removing pets for FA prevention.
Prenatal/postnatal environment	Saulyte et al. (2014) [81]	196 studies in 139 articles; active/passive smoke exposure vs. no exposure.	Tobacco smoke exposure.	Allergic rhinitis, allergic dermatitis and FA; FA limited to 8 studies and often self-reported.	In children/adolescents, active smoking was associated with allergic rhinitis (RR 1.40) and dermatitis (RR 1.36); passive smoke with rhinitis (RR 1.09) and dermatitis (RR 1.06). Passive smoke was associated with FA in cohort studies only (RR 1.43, 95% CI 1.12–1.83).	Observational and heterogeneous; smoke avoidance is strong general health advice, with FA-specific evidence indirect/low.

Smoke and pollution should be treated differently from siblings, daycare or pets because they are modifiable harms rather than neutral ecological markers. Smoke and air pollution should instead be considered avoidable harms: tobacco exposure has been associated with allergic diseases, including food allergy, while air pollution may contribute to epithelial injury, oxidative stress and allergic inflammation, although FA-specific evidence remains less consistent [34,81,82]. Overall, these exposures should be considered within a broader environmental framework rather than as established, independently modifiable strategies for FA prevention.

#### 3.3.6. Implementation, Caregiver Knowledge and Equity

Even strong evidence fails without implementation. Surveys in the United States found high awareness of peanut prevention guidance among allergists and paediatricians, but full implementation was far lower; barriers included uncertainty about applying the guideline, parental fear and difficulty arranging supervised feeds [83,84].

In a recent SIAIP caregiver survey on paediatric IgE-mediated FA, only a minority of caregivers considered themselves very informed, and recognition of reaction symptoms and anaphylaxis management was limited [85]. An SIAIP policy framework on anaphylaxis inequities further emphasised that access, education, emergency preparedness and regional variability must be considered part of prevention, not an afterthought [86].

Accordingly, implementation should be monitored through practical indicators. These include written counselling before the allergen introduction window, documentation of eczema severity, referral timing, safe food forms and frequency of continued ingestion. Other indicators include breastfeeding-compatible management, avoidance of unnecessary elimination, vaccination adherence, antimicrobial stewardship and regional access to specialist support.

## 4. Discussion

This review supports a prevention model that is organised across prenatal, perinatal and postnatal windows, but weighted according to the strength and clinical usability of the evidence. The prenatal period is relevant because maternal diet, nutritional status, metabolic health, environmental exposures and antimicrobial use may influence immune development, epithelial barrier function and early microbial programming. However, most prenatal exposures remain supported mainly by observational, mechanistic or indirect evidence rather than by consistent trial-level data showing prevention of challenge-confirmed food allergy. This distinction is clinically important: pregnancy counselling should promote nutritional adequacy, dietary diversity, avoidance of unnecessary restriction and correction of documented deficiencies, but should not transform plausible immunological pathways into prescriptive anti-allergy interventions.

The perinatal period adds a second layer of complexity. Mode of delivery, prematurity, neonatal intensive care, intrapartum or early-life antibiotic exposure and early feeding conditions may all shape microbial colonisation and immune maturation. Nevertheless, many of these factors are either medically indicated, non-modifiable, or ethically inappropriate targets for allergy prevention alone. Caesarean delivery, for example, may be associated with altered microbial acquisition, but obstetric safety must remain the overriding priority. Similarly, antibiotics should be used judiciously, but never withheld when clinically required. The value of including these factors in a prevention framework is therefore not to generate blame or unrealistic prescriptions, but to improve risk documentation, identify infants who may benefit from a closer follow-up and avoid unnecessary disruptions to breastfeeding or early nutrition.

The strongest actionable evidence remains concentrated in the postnatal period, particularly in infants with early eczema or other markers of increased allergic risk. In these children, prevention is no longer an abstract prenatal concept but a practical sequence: early recognition of eczema, active control of skin inflammation, protection of the epithelial barrier, avoidance of unnecessary food elimination and timely introduction of peanut and well-cooked egg when developmentally appropriate. The rationale for introducing well-cooked or baked egg is also supported by immunological considerations. Heating can denature heat-labile conformational epitopes of egg proteins and may reduce their allergenicity, potentially allowing safer exposure to egg allergens while supporting the development of oral tolerance [87]. This approach shifts prevention away from maternal or infant restriction and toward supervised, sustained dietary inclusion. It also recognises that delayed introduction in high-risk infants may miss a critical window in which oral exposure can support tolerance.

Breastfeeding requires a similarly balanced interpretation. Human milk provides immunological, nutritional and microbial signals that may support infant immune development, but the evidence does not justify using breastfeeding as a stand-alone food allergy prevention strategy or imposing maternal elimination diets for non-specific infant symptoms. The practical message is therefore protective rather than restrictive: breastfeeding should be supported, maternal diet should remain nutritionally complete whenever possible and elimination diets should be reserved for clear diagnostic or therapeutic indications, with dietetic supervision and planned reintroduction.

Several exposures frequently raised by families, including pets, siblings, daycare, vaccines, tobacco smoke, air pollution and household ecology, should be discussed without oversimplification. Siblings, pets and daycare may act as markers of microbial richness or household immune environment, but they cannot be converted into recommendations to engineer family structure or infection exposure. Vaccination should be maintained according to routine schedules; vaccine delay has no role as a food allergy prevention strategy. In contrast, tobacco smoke and avoidable pollutants are modifiable harms, and reducing exposure is justified by broad child health benefits even when food-allergy-specific estimates are less consistent.

The resulting prevention framework should therefore be non-restrictive, non-blaming and operational. It should help clinicians identify what can be recommended now, what should be monitored as contextual risk and what remains investigational.

On this basis, the SIAIP Commission for the Prevention of Allergic Diseases proposes a practical decalogue intended to support evidence-informed counselling during pregnancy, lactation and the first 1000 days of life. It is presented as a Commission-proposed clinical framework, not as a formal guideline or consensus statement pending a graded consensus process. The decalogue does not propose a single preventive intervention; rather, it integrates maternal nutrition, breastfeeding-compatible management, eczema control, safe allergen introduction, vaccination, environmental health and timely referral into a coherent clinical pathway. Its purpose is to support consistent counselling while preserving the central principle of prevention in paediatric allergy: reduce avoidable risk without creating unnecessary fear, restriction or delay in care. Table 2 links each statement to its target population, supporting references, expected benefit, potential harms and pragmatic strength. Table 2.

**Table 2:** Evidence to statement framework for the Commission-proposed clinical decalogue.

Statement	Target Population	Supporting References	Evidence Certainty/Directness	Expected Benefit	Potential Harm or Caution	Pragmatic Strength
1. Do not prescribe maternal avoidance of tolerated allergenic foods for primary prevention.	Pregnant and lactating women tolerating cow’s milk, egg, peanut, wheat, fish or other allergens.	[13,14,15,16,24,26]	Moderate; guidelines and systematic reviews; direct evidence does not support maternal avoidance for IgE-mediated FA prevention.	Prevents unnecessary restriction, nutritional inadequacy and maternal guilt.	Do not apply to structured diagnostic elimination for a symptomatic infant.	Strong against routine avoidance.
2. Promote a varied, nutritionally adequate maternal diet; present Mediterranean-style advice as general health/nutritional guidance, not proven FA prophylaxis.	Pregnant and lactating women.	[16,24,25,26,27,28,29,30]	Low for challenge-confirmed FA prevention; mostly observational and often indirect/composite allergic outcomes.	Supports maternal-child nutrition and may favour healthier immune/metabolic context.	Avoid implying that diet pattern alone prevents FA.	Good practice/conditional.
3. Correct documented nutritional deficiency or low intake, but do not use vitamin D, omega-3, probiotics, prebiotics or synbiotics as universal FA prophylaxis.	Pregnant women, lactating women and infants with nutritional risk, low omega-3 intake, increased allergic risk context or documented deficiency.	[13,14,15,24,36,38,39,40,41,42,44]	Low to very low for FA prevention; stronger rationale for nutritional adequacy or selected respiratory/inflammatory outcomes than for clinical FA.	Prevents deficiency and avoids replacing proven strategies with supplements.	Unnecessary supplementation, cost, false reassurance and variable product quality.	Strong against universal prophylaxis; conditional for deficiency correction or specialist-supervised risk-stratified use.
4. Use antimicrobial stewardship: avoid unnecessary antibiotics, but never withhold clinically indicated treatment.	Pregnancy, delivery, neonates and infants.	[18,52]	Low for FA-specific prevention; observational associations with confounding by indication.	Reduces avoidable antibiotic exposure and supports broader antimicrobial stewardship.	Undertreatment of maternal or neonatal infection would be harmful.	Good practice/contextual.
5. Record caesarean delivery, prematurity, NICU care and early colonisation as contextual factors, not modifiable FA prevention targets.	Infants with relevant perinatal history.	[18,46,53]	Low; mainly observational or mechanistic.	Improves risk documentation and follow-up planning.	Do not alter delivery decisions, withhold indicated care, use vaginal seeding or prescribe microbiome testing for FA prevention.	Contextual; no direct preventive recommendation.
6. Protect breastfeeding and avoid stopping breastfeeding or imposing broad maternal elimination for non-specific infant symptoms.	Breastfed infants and lactating mothers.	[13,14,15,55,56,57,58,59,62]	Moderate for breastfeeding support as general health guidance; low/inconsistent for FA prevention; mechanistic evidence indirect.	Supports breastfeeding continuation and maternal nutritional adequacy.	True reproducible reactions require structured diagnostic evaluation and planned reintroduction.	Strong for breastfeeding support; strong against unstructured elimination.
7. Recognise eczema early, treat established eczema actively and distinguish this from routine prophylactic emollient use.	Infants with early, persistent or moderate to severe eczema.	[13,14,18,64,65,66]	High for eczema as an FA risk marker; low/uncertain for emollients as FA prevention.	Links eczema care with allergy risk recognition and feeding planning.	Do not claim that eczema treatment alone has independently proven FA prevention; routine emollient prophylaxis is not supported.	Strong for eczema treatment and risk recognition; against universal emollient prophylaxis.
8. Provide written, food-specific and culturally feasible complementary feeding counselling before the allergen introduction window.	Families before and during complementary feeding, especially infants with eczema or caregiver anxiety.	[13,14,15,72,74,83,84]	Guideline-supported; implementation evidence shows gaps in uptake.	Improves timing, safe food form, dose regularity and adherence.	Vague advice may delay exposure or increase fear; whole nuts and unsafe textures must be avoided.	Strong implementation statement.
9a. Introduce peanut in safe, non-choking forms during the complementary feeding window, with regular continued ingestion after tolerance is confirmed.	Infants with severe eczema and/or egg allergy need risk assessment; infants with mild to moderate eczema or no eczema may introduce peanut according to age, readiness and local practice.	[5,6,7,13,14,15,72,73,74]	High for peanut in key RCTs/meta-analysis; guideline-based risk stratification.	Reduces IgE-mediated peanut allergy, especially in high-risk infants.	Prior immediate reaction, known peanut allergy or severe uncontrolled eczema should prompt allergy assessment. Use smooth/diluted peanut; avoid whole nuts. Continue regular intake, approximately 6–7 g peanut protein/week over at least 3 feedings when feasible.	Strong for peanut allergy prevention with safety pathways.
9b. Introduce well-cooked egg during complementary feeding; avoid raw or lightly cooked egg as a prevention strategy.	Infants who are developmentally ready for complementary feeding, including those at general or increased allergic risk.	[8,13,14,15,72,74,87]	Moderate to high for cooked egg; direct egg allergy prevention evidence stronger than for most other foods.	Reduces IgE-mediated egg allergy when introduced in tolerated cooked forms and continued.	Raw or insufficiently cooked egg is not the preventive strategy and may be less tolerated.	Strong/moderate to strong for well-cooked egg introduction.
9c. Introduce milk, wheat and other tolerated allergens as part of a varied complementary diet, without presenting them with the same certainty as peanut and egg.	Infants developmentally ready for complementary feeding and without previous immediate reaction to the specific food.	[8,13,14,15,65,72,74,75,76,77,78,79]	Conditional; multiple food evidence is less food-specific and more adherence-dependent.	Supports dietary diversity and may reduce overall IgE-mediated FA risk.	Avoid forced intake and unnecessary restriction; suspected immediate reactions require clinical assessment.	Conditional/expert-informed; dietary diversity guidance rather than proven prophylaxis for every allergen.
10a. Maintain routine vaccination schedules; do not delay vaccines for allergy prevention.	All infants.	[13,14,15,63]	Moderate for no allergy increase signal; not an FA prevention intervention.	Protects child health and prevents vaccine delay.	Vaccine delay has no role in FA prevention.	Strong against delay.
10b. Reduce modifiable environmental harms, especially tobacco smoke and avoidable pollutants, while avoiding fear-based counselling.	Pregnant women, infants and households.	[32,34,81]	Low for FA-specific prevention; stronger for broad respiratory and epithelial barrier health.	Reduces avoidable environmental harm and supports general child health.	Do not overstate FA-specific causal prevention.	Strong for general child health; contextual for FA.
10c. Interpret siblings, daycare, pets and household ecology as contextual exposures, not prescriptions; monitor implementation and equity indicators.	Infants and families receiving prevention counselling.	[34,48,80,83,84,85,86,88]	Low for ecological exposures; implementation/equity evidence is survey or policy based.	Avoids inappropriate manipulation of family environment and improves counselling delivery.	Do not advise acquiring/removing pets or engineering infections for FA prevention.	Contextual/implementation good practice.

As members of the SIAIP Commission on Primary and Secondary Prevention of Allergic Diseases, we contributed to the development of an educational booklet aimed at disseminating evidence-based recommendations on measures to help prevent allergic diseases across the different stages of childhood [88].

## 5. Limitations and Future Directions

This review is narrative rather than systematic, and study selection was not conducted with a registered protocol. As a narrative review, it may also be subject to selection bias, and studies were not screened independently or in duplicate. We did not perform a formal risk of bias assessment or apply a formal evidence-grading framework. The highest certainty evidence concerns infant feeding, not maternal intervention. Maternal dietary pattern studies are observational and often use heterogeneous scores, parent-reported outcomes or composite allergic endpoints rather than oral-food-challenge-confirmed FA. The heterogeneity in FA definitions and outcome ascertainment across studies further limits direct comparison and interpretation. Mechanistic studies on IgG immune complexes, SCFAs, HMOs and microbiota are biologically important but often rely on animal models, small samples or surrogate endpoints. The evidence base is also weighted toward European and other high-income settings, limiting generalisability to populations with different dietary patterns, cultural practices, environmental exposures and healthcare resources. In particular, cultural feasibility and allergen introduction practices may differ substantially across countries and healthcare systems. Finally, translating observational associations into clinical or policy recommendations remains challenging because associations may reflect residual confounding, reverse causation or differences in healthcare access and adherence. These limitations are particularly relevant to the policy-oriented nature of this review and should be considered when interpreting the proposed recommendations.

Future research should prioritise prenatal whole-diet interventions with predefined challenge-confirmed FA outcomes; harmonised measurement of maternal and infant diet diversity; longitudinal maternal–infant microbiome and metabolome sampling; standardised human milk immune and HMO profiling; and pragmatic trials that measure adherence, safety, acceptability and equity. Future studies should also aim to improve the representation of diverse populations and healthcare settings and to evaluate whether prevention strategies are culturally feasible and transferable across different countries and health systems. Perinatal studies should separate birth mode, labour exposure, antibiotics, prematurity and neonatal care intensity rather than treating caesarean delivery as a single causal exposure.

For SIAIP, the next step should be a formally graded consensus process accompanied by a one-page counselling tool, explicit referral criteria, dietetic safeguards for maternal elimination, standardised eczema severity prompts, vaccination reassurance, antimicrobial stewardship language and a national minimum dataset covering age at peanut and egg introduction, breastfeeding continuation, caregiver confidence and regional specialist access.

## 6. Conclusions

The primary prevention of IgE-mediated FA during the first 1000 days should be framed around a limited number of actionable clinical messages rather than as a broad causal pathway. The best-supported actions are to avoid unnecessary maternal or infant dietary restriction, identify infants at increased risk through early or moderate to severe eczema and relevant allergy history, and implement safe, developmentally appropriate and sustained introduction of peanut and well-cooked egg once tolerated. Breastfeeding support, maternal diet quality, nutritional adequacy, vaccination, smoke avoidance and antimicrobial stewardship remain important for maternal and child health and for avoiding preventable harm, but they should not be presented as stand-alone, proven FA prevention interventions. Maternal age, caesarean delivery, prematurity, siblings, daycare and pet exposure should be documented as contextual risk markers rather than modifiable prescriptions, while microbiome profiles, HMOs and microbial supplementation remain investigational. The practical aim of the SIAIP framework is therefore non-restrictive, non-blaming counselling that creates safe opportunities for oral tolerance without overstating causality.

## Data Availability

No new data were created or analyzed in this study. Data sharing is not applicable to this article.
